# Capsaicin Inhibited Aggressive Phenotypes through Downregulation of Tumor-Associated NADH Oxidase (tNOX) by POU Domain Transcription Factor POU3F2

**DOI:** 10.3390/molecules21060733

**Published:** 2016-06-04

**Authors:** Hung Yen Chen, Yi Hui Lee, Huei Yu Chen, Chia An Yeh, Pin Ju Chueh, Yi-Mei J. Lin

**Affiliations:** Institute of Biomedical Sciences, National Chung Hsing University, Taichung 40227, Taiwan; p311308@hotmail.com (H.Y.C.); cornsugar@gmail.com (Y.H.L.); trista09081123@gmail.com (H.Y.C.); r.leafage000@gmail.com (C.A.Y.); pjchueh@dragon.nchu.edu.tw (P.J.C.)

**Keywords:** POU3F2-binding motif, tumor-associated NADH oxidase (tNOX), transcriptional regulation, cancer

## Abstract

Capsaicin has been reported to preferentially inhibit the activity of tumor-associated NADH oxidase (tNOX), which belongs to a family of growth-related plasma membrane hydroquinone oxidases in cancer/transformed cells. The inhibitory effect of capsaicin on tNOX is associated with cell growth attenuation and apoptosis. However, no previous study has examined the transcriptional regulation of tNOX protein expression. Bioinformatic analysis has indicated that the tNOX promoter sequence harbors a binding motif for POU3F2, which is thought to play important roles in neuronal differentiation, melanocytes growth/differentiation and tumorigenesis. In this study, we found that capsaicin-mediated tNOX downregulation and cell migration inhibition were through POU3F2. The protein expression levels of POU3F2 and tNOX are positively correlated, and that overexpression of POU3F2 (and the corresponding upregulation of tNOX) enhanced the proliferation, migration and invasion in AGS (human gastric carcinoma) cells. In contrast, knockdown of POU3F2 downregulates tNOX, and the cancer phenotypes are affected. These findings not only shed light on the molecular mechanism of the anticancer properties of capsaicin, but also the transcription regulation of tNOX expression that may potentially explain how POU3F2 is associated with tumorigenesis.

## 1. Introduction

Capsaicin (8-methyl-*N*-vanillyl-6-nonenamide), an important constituent of chili peppers, has emerged as a chemopreventative agent owing to its anti-growth activity against various cancer cell systems [1,2,3,4]. Interestingly, capsaicin is shown to inhibit tNOX activity in transformed cancer cells, leading to attenuated cell growth through apoptosis, while leaving non-cancerous cells undamaged [1]. Furthermore, capsaicin exposure instigates tNOX suppression accompanied by apoptosis, and tNOX knockdown by RNA interference enhances stress-induced apoptosis in cancer cells [5,6]. On the other hand, cell proliferation and migration are reported to be augmented by low concentrations of capsaicin through transient tNOX upregulation [7]. However, the transcriptional regulation of tNOX protein has not yet been studied before.

Transcription factors respond to specific signals of particular biological systems by modulating downstream protein targets and cellular functions. Therefore, it is important to characterize the transcriptional targets of these factors and to study their regulatory machineries. The POU domain, class 3, transcription factor 2 (POU3F2; also known as Brn2 or N-Oct-3) belongs to a large family of brain-specific homeobox transcription factors that bind to an octameric DNA sequence. The *POU3F2* gene is expressed in the central nervous system (CNS) during neuronal development and in adult brain [8]. POU3F2 has been shown to form a transcriptional regulatory complex by interacting with various proteins, including: itself (via homo-dimerization); TATA binding protein (TBP); the transcriptional coactivator, p300; Sox-10 in melanocytic regulation [9]; and Jab1, whose encoded gene has been related to neurodegenerative diseases [10]. POU3F2 has been proposed to participate in modulating several important CNS-related genes, and the evidence clearly supports the involvement of this transcription factor in diverse neuronal functions. For example, POU3F2 has been shown to regulate the expression levels of critical genes at different stages of neural differentiation [11,12], the migration of cortical neuron [13], the neurogenesis and positioning of cortical neurons [14,15], *etc*. Moreover, Pou3f2-knockout mouse studies have demonstrated that the protein plays important roles in determining neuronal lineages in the hypothalamus and regulating the development/survival of the endocrine hypothalamus and posterior pituitary gland [16,17].

In addition to the abilities of POU3F2 to regulate neuron differentiation, accumulating evidence suggests that it is also involved in different types of cancer. For example, oncogenic BRAF has been shown to support the proliferation and survival of melanoma cells through the upregulation of POU3F2 and MITF [18], and POU3F2 expression appears to be important for the proneural/neuroendocrine differentiation of lung cancer cells [19,20]. However, although POU3F2 seems to be important for cancer progression, little is known about the downstream genes and underlying mechanisms involved in these tumorigenic effects.

Here, we examined the association between POU3F2 and tNOX expression in stomach cancer cells. Based on our novel results, for the first time, we report that capsaicin reduced cancer phenotypes through POU3F2-medaited tNOX downregulation, and overexpression of POU3F2 in AGS cells enhances tNOX protein expression that is associated with increased cell proliferation and migration.

## 2. Results

### 2.1. Capsaicin Suppressed tNOX Expression and Cell Migration

**Figure 1:**
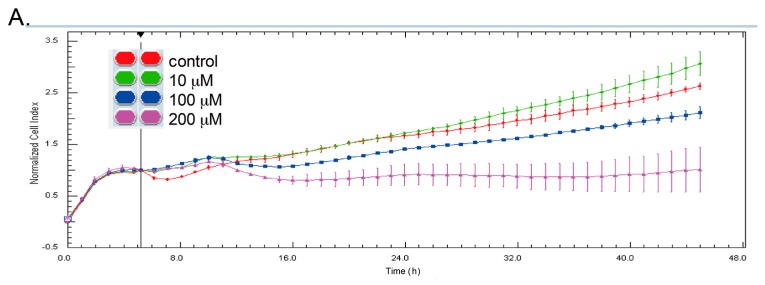

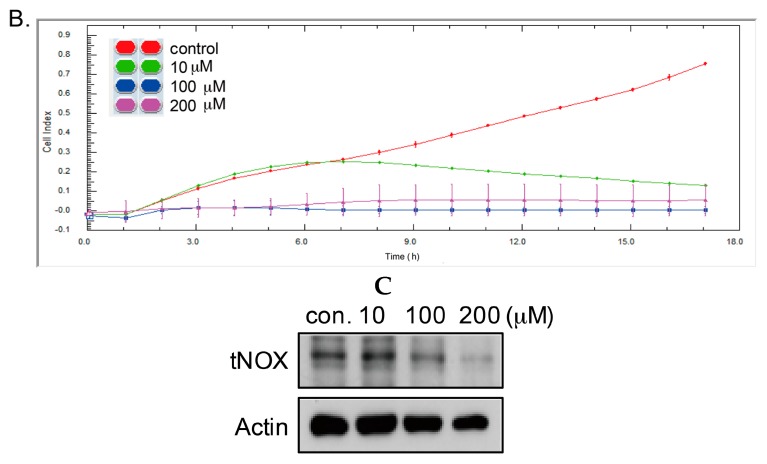
Capsaicin inhibits cell proliferation, and migration is associated with tNOX downregulation. (**A**) Cell growth was dynamically monitored using impedance technology in AGS cells; (**B**) Cell migration was dynamically monitored using impedance technology in AGS cells; (**C**) Cell lysates were separated by SDS-PAGE and analyzed by Western blotting. β-Actin was used as an internal control. con., control.

The inhibitory effect of capsaicin was examined in gastric cancer AGS cells in this study. The dynamic effect of capsaicin on cell growth was continuously monitored by cell impedance measurements, displaying the results as cell index values [21]. We found that AGS cell growth was inhibited by capsaicin at 100 and 200 µM (Figure 1A). We next examined the effect of capsaicin on cell migration utilizing the cell impedance measurements. Capsaicin successfully diminished cell migration in AGS cells at the concentrations tested (Figure 1B). Since protein levels of tNOX are positively associated with cell proliferation and migration [7,22], we also analyzed the effect of capsaicin on tNOX expression. Consistent with the inhibitory effect of capsaicin on cellular functions, our results demonstrated that tNOX was downregulated by capsaicin at 100 and 200 µM (Figure 1C). These lines of evidence suggest that tNOX downregulation is correlated with capsaicin-reduced cancer phenotypes in AGS cells.

### 2.2. The Expression of tNOX is Transcriptionally Regulated by POU3F2 in Human Stomach Cancer Cells

**Figure 2:**
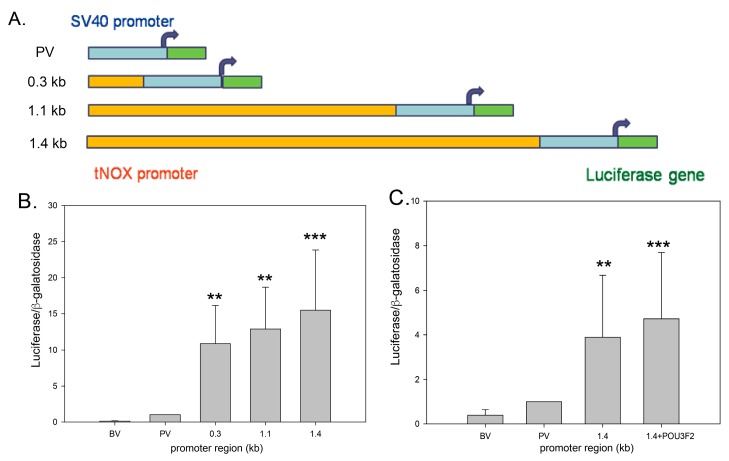
*tNOX* as a potential target gene for POU3F2. (**A**) Schematic showing the different lengths of the *tNOX* promoter region used in our luciferase assays; (**B**) AGS cells were transfected with reporter constructs containing different lengths of the *tNOX* promoter region, and luciferase activities were determined. The presented values (mean ± SD) represent three independent experiments performed in at least triplicate (** *p* < 0.01, *** *p* < 0.001 for experimental groups *vs.* positive controls); (**C**) Cells were transfected with reporter constructs of the 1.4-kb *tNOX* promoter region or co-transfected with the POU3F2 expression vector, and luciferase activities were determined. The presented values (mean ± SD) represent three independent experiments performed in at least triplicate (** *p* < 0.01, *** *p* < 0.001 for experimental groups *vs.* positive controls).

Bioinformatic analyses identified several potential POU3F2 binding sites in the promoter region of *tNOX*. To assess whether *tNOX* is a direct transcriptional target of POU3F2, we performed luciferase reporter assays using different lengths of the 5′promoter region of the human *tNOX* gene. The fragments were subcloned into the 5′promoter region of the pGL3-Basic vector to construct a series of recombinant promoter-luciferase reporters for exploring the regulating effects of different promoter elements (Figure 2A). The assessment of luciferase activities in AGS human stomach cancer cells revealed that pGL3-1.4 kb exhibited the highest luciferase activity among the tested constructs (Figure 2B). When pGL3-1.4 kb was co-transfected with a POU3F2-expressing plasmid, the luciferase activity was further increased, although the difference was not significant due to the masking effect of endogenous POU3F2 expression (Figure 2C). This suggested that POU3F2 could be a potential transcription factor for tNOX expression.

**Figure 3:**
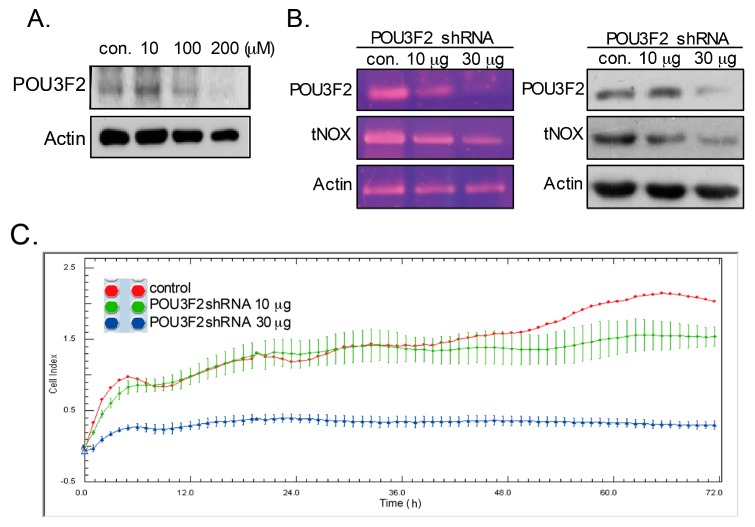
Capsaicin-suppressed POU3F2 and shRNA-mediated knockdown of POU3F2 reduces tNOX expression and suppresses the growth of AGS cancer cells. (**A**) Cells were exposed to capsaicin, and cell lysates were separated by SDS-PAGE and analyzed by Western blotting. β-actin was used as an internal control; (**B**) Cells were transfected with shRNA-targeted POU3F2 for 24 h and harvested for protein analyses. Cell lysates were separated by SDS-PAGE and analyzed by Western blotting. β-actin was used as an internal control. The mRNA levels of *POU3F2* and *tNOX* were determined by RT-PCR using β-actin as an internal control; (**C**) AGS cell growth was dynamically monitored using impedance technology. Normalized cell index (CI) values measured over 72 h are shown. con., control.

To further investigate the correlation between POU3F2 and capsaicin-mediated tNOX downregulation, the effect of capsaicin on POU3F2 expression was studied. Our results demonstrated that capsaicin efficiently inhibited POU3F2 expression (Figure 3A). We also performed small hairpin RNA (shRNA)-mediated gene silencing of POU3F2. The mRNA and protein expressions of POU3F2 were markedly and specifically reduced in AGS cells treated with the POU3F2-targeting shRNAs, and these cells also exhibited both transcriptional and translational downregulation of tNOX expression (Figure 3B). Consistent with the previous association of tNOX expression with cancer cell growth [5,22], cell impedance measurements revealed that POU3F2 knockdown cells exhibited reduced cell growth compared to control cells (Figure 3C). Thus, our results indicate that tNOX expression is positively correlated with the level of POU3F2 and that the POU3F2 depletion-mediated downregulation of tNOX is associated with the reduced cell growth of AGS cells.

### 2.3. POU3F2 Overexpression Upregulates tNOX Expression and Increases Cell Proliferation

**Figure 4:**
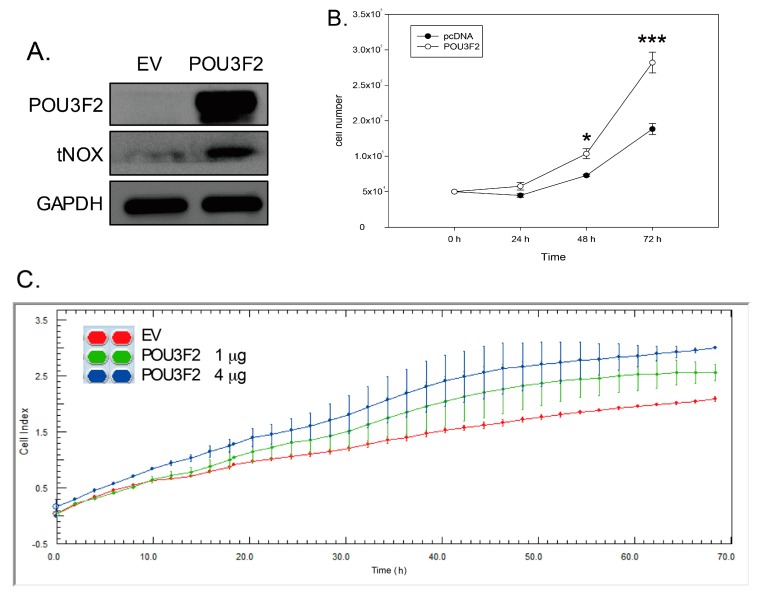
The overexpression of POU3F2 enhances tNOX expression and increases the growth of AGS cancer cells. (**A**) Cell lysates were separated by SDS-PAGE and analyzed by Western blotting. GAPDH was used as an internal control; (**B**) AGS cells were seeded at least in triplicate, and the number of viable cells was determined by trypan blue exclusion assays. The presented values (mean ± SD) represent three independent experiments performed in at least triplicate (* *p* < 0.05, *** *p* < 0.001 for experimental groups *vs.* controls); (**C**) AGS cell growth was dynamically monitored using impedance technology. Normalized CI values measured over 72 h are shown.

We next utilized a gain-of-function approach to confirm that POU3F2 regulates tNOX expression. Consistent with this model, overexpression of POU3F2 in AGS cells was associated with upregulation of tNOX (Figure 4A); significantly increased cell proliferation, as analyzed by trypan blue exclusion (Figure 4B); and increased cell growth, as assessed by cell impedance measurements (Figure 4C). 

### 2.4. POU3F2-Mediated tNOX Upregulation Shortens the Cell Doubling Time

**Figure 5:**
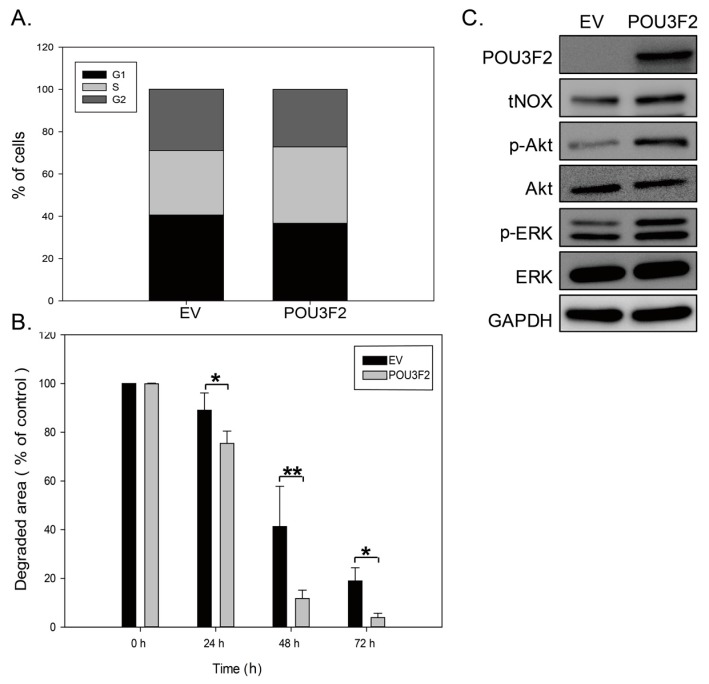
The overexpression of POU3F2 increases AGS cell proliferation. (**A**) AGS cells were assayed for cell cycle phase. The graphs are representative of three independent experiments. Values (mean ± SD) are from three independent experiments. There was no significant difference among the groups; (**B**) Cell division was analyzed by 5-chloromethylfluorescein diacetate (CMFDA) staining of POU3F2-over expressing and control cells. The presented values (mean ± SD) represent at least three independent experiments (* *p* < 0.05, ** *p* < 0.01 for POU3F2-overexpressing cells *vs.* controls); (**C**) Cell lysates were separated by SDS-PAGE and analyzed by Western blotting. GAPDH was used as an internal control.

To expand on our observations, we conducted cell cycle analyses. Our results demonstrated that the cell populations in each phase of the cell cycle did not significantly differ between POU3F2 overexpressing and control cells (Figure 5A). However, staining with 5-chloromethylfluorescein diacetate (CMFDA, a tracking dye for cell division) showed that the POU3F2-mediated upregulation of tNOX decreased the doubling time (Figure 5B). The cell doubling time as calculated using the Real-Time Cell Analyzer (RTCA) system also indicated a shortened cell cycle progression. Furthermore, Western blot analyses showed that phosphorylated/activated ERK and Akt, important for cell proliferation, were enhanced in POU3F2-mediated tNOX upregulated cells compared to controls (Figure 5C). These findings support the notion that the POU3F2-mediated enhancement of tNOX expression increases cell proliferation (Figure 4).

### 2.5. POU3F2-Mediated tNOX Upregulation Enhances Cell Migration and Invasion

**Figure 6:**
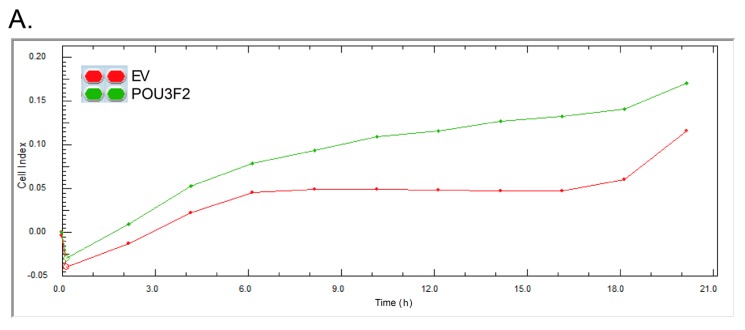

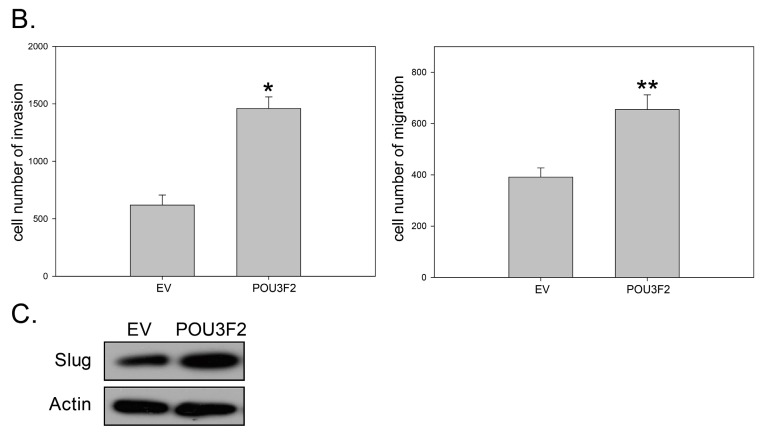
The overexpression of POU3F2 increases AGS cell migration and invasion. (**A**) Dynamic monitoring of AGS cell migration was performed using impedance technology. Normalized CI values obtained over 20 h are presented; (**B**) Cell migration and invasion were measured by Boyden chamber system. Values (mean ± SD) are from at least three independent experiments. Cell migration was significantly enhanced in POU3F2-overexpressing cells compared to controls (* *p* < 0.05). Cell invasion was significantly increased in POU3F2-overexpressing cells compared to controls (** *p* < 0.01); (**C**) Cell lysates were separated by SDS-PAGE and analyzed by Western blotting. β-actin was used as an internal control.

Because tNOX expression has been positively associated with cell migration, we further investigated whether the POU3F2-overexpression altered the migration of AGS cells. Cell migration was monitored by electrical impedance measurements, which are generated by the presence of migratory cells on the top of an electrode, and is presented as cell index (CI) values [23,24]. Our results revealed that overexpression of POU3F2 enhanced cell migration, as assessed by cell impedance measurement (Figure 6A) and Boyden chamber assays (Figure 6B). POU3F2-overexpressing cells also exhibited increased expression of Slug (Figure 6C), which is a transcription factor suppressing E-cadherin expression and increasing cell invasion and the epithelial-mesenchymal transition (EMT) [25,26].

## 3. Discussion

In this study, we use AGS gastric cancer cells as a model to clarify the relationship between capsaicin-reduced cancer phenotypes and tNOX expression. We identify tNOX as a new downstream target of POU3F2 and provide evidence that their positive relationship may explain the pro-tumor functions of POU3F2 in promoting cell proliferation, migration and invasion.

Recent work has focused on the association of POU3F2 expression with tumorigenesis, particularly aggressive cancer cell phenotypes [27,28,29,30]. Through its ability to modulate MAPK pathway activation and MITF expression, POU3F2 can suppress melanocytes differentiation and increase tumor metastasis [31]. Moreover, POU3F2 was reported to transcriptionally repress T-cadherin, which is a tumor suppressor that is expressed at extremely low levels in several types of carcinomas [32]. POU3F2 and other neuro-developmental transcription factors (SOX2, SALL2 and OLIG2) coordinate to play essential roles in the progression of glioblastoma [30]. In small cell lung cancer lines, both gain-of-function and loss-of-function strategies have shown that POU3F2 regulates the expression of achaete-scute homolog-like 1 (ASCL1), which functions as a growth enhancer and helps tumor cells escape anti-cancer immune responses [20]. Together, these previous findings support the idea that the transcriptional targets of POU3F2 contribute to aggressive cancer phenotypes. 

Tumor-associated NADH oxidase (tNOX) belongs to a family of growth-related plasma membrane hydroquinone oxidases that are responsible for converting reduced NADH to the oxidized NAD^+^ form [33]. This NADH oxidase activity was also detected in pooled sera from cancer patients, but not healthy volunteers, suggesting that tNOX could be an available target for cancer research [34,35,36]. Indeed, RNA interference-mediated knockdown of tNOX was found to impair the cell proliferation and migration of human cervical carcinoma HeLa cells [22], whereas overexpression of tNOX caused non-cancerous MCF-10A cells to acquire aggressive phenotypes, such as enhanced invasiveness [37]. More interestingly, transit upregulation of tNOX by capsaicin or anticancer agents has been shown to enhance EMT, suggesting that tNOX plays an essential role in cancer phenotypes [7,38]. As no previous study had addressed the transcriptional regulation of tNOX, it is notable that we herein show for the first time that the transcription factor, POU3F2, may regulate tNOX expression. Moreover, we show that the expression levels of POU3F2 and tNOX are correlated and that changes in their levels can alter the proliferation, migration and invasion of AGS gastric cancer cells. 

Taken together, our results show that overexpression of POU3F2 in AGS cells induces the upregulation of tNOX and the acquisition of aggressive cancer phenotypes, including increased cell growth, migration and invasion. In contrast, capsaicin-suppressed POU3F2 or knockdown of POU3F2 both downregulates tNOX and affects the cancer phenotypes. These findings further suggest the plausible scenario wherein the pro-tumor role of POU3F2 reflects its ability to positively regulate tNOX expression.

## 4. Materials and Methods

### 4.1. Materials

Fetal bovine serum (FBS) and penicillin/streptomycin were obtained from Gibco/BRL Life Technologies (Grand Island, NY, USA). The anti-phospho-ERK, anti-ERK, anti-phospho-Akt, and anti-Akt antibodies were purchased from Cell Signaling Technology, Inc. (Beverly, MA, USA). The anti-Slug antibody and anti-POU3F2 antibody was purchased from Santa Cruz Biotechnology, Inc. (Santa Cruz, CA, USA). The anti-β-actin antibody was from Millipore Corp. (Temecula, CA, USA). The anti-GAPDH antibody was purchased from GeneTex, Inc. (Irvine, CA, USA). The antisera to tNOX used in Western blot analyses were generated as described previously [39]. The anti-mouse and anti-rabbit IgG antibodies and other chemicals were purchased from Sigma Chemical Company (St. Louis, MO, USA), unless specified otherwise.

### 4.2. Cell Culture and Transfection

AGS cells (human gastric adenocarcinoma cells derived from human stomach cancers) were kindly provided by Chun-Ying Wu (Department of Gastroenterology, Taichung Veterans General Hospital, Taiwan). The cells were grown in RPMI-1640 supplemented with 10% FBS, 100 units/mL penicillin and 50 µg/mL streptomycin at 37 °C in humidified air containing 5% CO_2_, and the medium was replaced every 2–3 days.

The POU3F2-specific and negative control RNA interference were constructed at the RNAi core facility at Academic Sinica (Taipei, Taiwan). Briefly, cells were seeded in 10-cm dishes, allowed to attach overnight and then transfected with POU3F2 shRNA or control shRNA using Lipofectamin RNAiMAX Reagent (Life Technologies) according to the manufacturer’s instructions.

### 4.3. Plasmid Constructs and Luciferase Assay

The full protein-encoding sequence of the *POU3F2* gene was amplified from human cDNA with POU3F2_CDSF_BamHI and POU3F2_CDSR_XbaI, which were designed to introduce *BamHI* and *XbaI* sites, respectively, at their 5′ ends. The generated PCR products were digested with *HindIII* and *XbaI* and cloned into the pCDNA3.1/Myc_His(+) A vector. 

Different lengths of the 5′-flanking DNA sequence of the *tNOX* gene were PCR amplified from the genomic DNA of HCT116 cells. The PCR products were subcloned into the pGL3-basic luciferase reporter vector (Promega, Madison, WI, USA) to generate constructs that were designated as pGL-0.3 kb, pGL-1.1 kb and pGL-1.4 kb. The reporter vectors plus the POU3F2 expression plasmid or empty vector were co-transfected into AGS cells using Lipofectamin2000 (Promega) according to the manufacturer’s instructions. Cells were harvested 48 h after transfection, and luciferase activity was measured using the Dual-Luciferase Reporter Assay System (Promega) according to the manufacturer’s instructions. 

### 4.4. Continuous Cell Monitoring with the Cell Impedance Measurement System

For continuous monitoring of changes in cell growth, cells (7.5 × 10^3^ per well) were seeded onto E-plates and incubated for 30 min at room temperature. The E-plates were placed onto the Real-Time Cell Analysis (RTCA) station (xCELLigence System, Roche, Mannhein, Germany), and the cells were grown overnight. For continuous monitoring of cell migration, cells (10^4^ per well) were seeded onto the top chamber of a cell invasion and migration (CIM) plate, which features microelectronic sensors integrated on the underside of the microporous polyethylene terephthalate (PET) membrane of a Boyden-like chamber. After incubation for 30min at room temperature, the CIM plates were placed onto the RTCA station. Cell migration was continuously monitored based on changes in the electrical impedance at the electrode/cell interface.

### 4.5. Trypan Blue Exclusion Assay

Cells were seeded at 10^4^ cells per dish, cultured for the indicated time periods, trypsinized, collected by centrifugation and washed with PBS. Cell pellets were collected, the cells were suspended in 50 µL PBS and 50 µL of 0.4% (*w*/*v*) trypan blue stain, and cell numbers were counted and recorded.

### 4.6. Cell Cycle Analysis 

In brief, 10^6^ cells were collected, washed in PBS, slowly fixed in 75% ethanol and kept at −20 °C for at least 1 h. The cell pellet was washed with PBS, centrifuged at 500× *g* for 5 min and resuspended in 200 µL cold PBS. Nuclear DNA was stained in the dark with a propidium iodide (PI) solution (20 mM Tris pH 8.0, 1 mM NaCl, 0.1% NP-40, 1.4 mg/mL RNase A, 0.05 mg/mL PI) for 30 min on ice. The total cellular DNA content was analyzed with a FC500 flow cytometer (Beckman Coulter Inc., Brea, CA, USA).

### 4.7. Cell Division Assay

CellTracker Green CMFDA (5-chloromethylfluorescein diacetate; Molecular Probes, Eugene, OR, USA) is a fluorescent dye that can be well absorbed and retained in living cells through several cell cycles. POU3F2-overexpressing and control AGS cells were labeled with 5 μM CMFDA in fresh medium for 45 min. The cells were then washed with PBS and trypsinized, and cell division was assessed by flow cytometry according to the previous publications [40,41].

### 4.8. Boyden Chamber Assay

A Boyden chamber with 8-µm pore filter inserts (Neuro Probe, Inc., Gaithersburg, MD, USA) was used to measure cell migration. Cells (2.5 × 10^3^) in RPMI-1640 containing 0.5% of FBS were placed in the upper chamber, and the lower chamber was filled with complete RPMI-1640. After 24 h in culture, the cells were fixed in methanol for 15 min and then stained with 10% Giemsa in PBS for 30 min. Cells on the upper side of the filters were removed with cotton-tipped swabs, and the filters were washed in PBS. The cells on the filter bottom (migrated cells) were counted and recorded. 

### 4.9. Western Blot Analysis 

Cell extracts were prepared in lysis buffer containing 20 mM Tris-HCl pH 7.4, 100 mM NaCl, 5 mM EDTA, 2 mM phenylmethylsulfonyl fluoride (PMSF), 10 ng/mL leupeptin and 10 µg/mL aprotinin. Equal amounts of proteins (40 µg) were applied to SDS-PAGE gels, and resolved proteins were transferred to nitrocellulose membranes (Schleicher & Schuell, Keene, NH, USA). The membranes were blocked with nonfat milk solution for 30 min and then washed and probed with primary antibodies. Membranes were rinsed with Tris-buffered saline containing 0.1% Tween 20 and incubated with horseradish peroxidase-conjugated secondary antibodies for 2 h. Finally, the membranes were rinsed and developed using enhanced chemiluminescence (ECL) reagents (Amersham Biosciences, Piscataway, NJ, USA).

### 4.10. Reverse Transcriptase-Polymerase Chain Reaction

Total RNA from gastric cancer cells was isolated using the TRIzol reagent (GIBCO, Carlsbad, CA, USA). First-strand cDNA was synthesized from 1 μg total RNA using Superscript II (Life Technologies, Rockville, MD, USA). The following primer sets were used for PCR amplifications: tNOX, 5’-GAAGTGTGATGCCGATAACAG-3’ (sense) and 5’-AGTACTAGAGCCCAGGCGAA-3’ (antisense); β-actin, 5′-ACTCACCTTGGTGGTGCATA-3’ (sense) and 5’-ACACCTTGATGGGAAAGGTGG-3’ (antisense); POU3F2, 5’-TGGGATTTACCCAAGCGGAC-3’ (antisense) and 5’-TGTGGTGGAGTGTCCCTACT-3’ (antisense). The reaction conditions consisted of 30 cycles of 95 °C for 1 min, 58 °C for 1 min and 72 °C for 1 min, followed by a final extension of 7 min at 72 °C. The PCR products were resolved by 0.8% agarose gel electrophoresis and visualized by ethidium bromide staining.

### 4.11. Statistics

All data are expressed as the mean ± SD of three or more independent experiments. Between-group comparisons were made by one-way analysis of variance (ANOVA) followed by an appropriate *post hoc* test. A value of *p* < 0.05 was considered to be statistically significant.
